# Metabolic profiling of the TME uncovers the contrasting impacts of CKMT2 and PDE2A in CRC progression and therapeutic response

**DOI:** 10.3389/fphar.2026.1732137

**Published:** 2026-03-09

**Authors:** Yuxiang Fu, Jianbo Lai, Kaibin Huang, Liping Liu, Guixiang Liao

**Affiliations:** 1 Department of Gastrointestinal Surgery, Shenzhen People’s Hospital, The Second Clinical Medical College, Jinan University, Shenzhen, China; 2 Department of Hepatobiliary and Pancreas Surgery, Shenzhen People’s Hospital, The Second Clinical Medical College, Jinan University, Shenzhen, China; 3 Department of Radiation Oncology, Shenzhen People’s Hospital (The Second Clinical Medical College, Jinan University, The First Affiliated Hospital, Southern University of Science and Technology), Shenzhen, China

**Keywords:** cancer hallmarks, colorectal cancer, metabolic reprogramming, RNA sequencing, tumor microenvironment

## Abstract

**Background:**

Colorectal cancer (CRC) remains a major cause of cancer-related morbidity and mortality, with high recurrence rates and limited treatment options for metastatic disease. The tumor microenvironment (TME) and metabolic reprogramming are critical drivers of CRC progression, influencing immune responses, therapeutic resistance, and patient outcomes.

**Objective:**

This study explores the interplay between metabolic reprogramming and the TME in CRC using transcriptomic data and bioinformatics approaches to identify metabolically and microenvironmentally defined CRC subtypes and candidate biomarkers.

**Methods:**

Gene expression and clinical data were obtained from TCGA colorectal adenocarcinoma (COAD), rectal adenocarcinoma (READ), and six GEO CRC datasets. Immunohistochemistry (IHC) was performed to validate PDE2A and CKMT2 expression in CRC tissues. Bioinformatic analyses were conducted using R software v4.0.3.

**Results:**

We identified 220 TME- and 40 metabolism-related differentially expressed genes (DEGs) in CRC. Consensus clustering of these TMET genes revealed two distinct subtypes: Cluster 1 (C1), associated with poorer survival, an immune-mesenchymal phenotype, and frequent mutations in TTN and BRAF, and Cluster 2 (C2), characterized by enriched TP53 and APC mutations, classic tumor suppressor pathway activation, and higher genomic instability. Metabolically, C1 was characterized by lipid metabolism and extracellular matrix remodeling, whereas C2 showed enrichment of nucleotide and amino acid metabolism linked to cell cycle progression and DNA repair. Single-cell RNA sequencing confirmed these distinctions, revealing that C1-upregulated genes were predominantly expressed in immune and stromal compartments, whereas C2-upregulated genes were enriched in epithelial and malignant cells. PDE2A, primarily expressed by endothelial cells, was identified as a metabolic biomarker of C1, while CKMT2, expressed in malignant cells, defined C2. These genes serve as key metabolic markers distinguishing CRC subtypes based on molecular heterogeneity and prognosis.

**Conclusion:**

PDE2A and CKMT2 were identified as critical metabolic biomarkers associated with distinct CRC subtypes and TME compositions. These findings highlight the intricate relationship between metabolic reprogramming, the tumor microenvironment, and tumor heterogeneity, providing insights into CRC molecular subtypes and their prognostic significance.

## Introduction

According to GLOBOCAN 2022, colorectal cancer (CRC) was the third most diagnosed cancer (9.6% of cases) and the second leading cause of cancer death (9.3%) worldwide (Bray et al., 2024). In China, it was the second most common cancer, with 517,100 new cases, and the fourth leading cause of cancer death, responsible for 240,000 deaths (Han et al., 2024). Therapeutic strategies for CRC include surgery, chemotherapy, and molecularly targeted therapies (Fadlallah et al., 2024). While these treatments can effectively manage localized disease, nearly 20% of patients have metastatic disease at presentation and another 25% of patients with localized CRC develop metastases (Biller and Schrag, 2021). Surgical resection, though a common approach, has a high recurrence rate, with 80% of patients experiencing progression within 3 years and 95% within 5 years (Obrand and Gordon, 1997; Seo et al., 2013). Only a small fraction of metastatic CRC patients (15%) are eligible for surgical intervention. For others, molecular therapies can offer prolonged survival, though challenges such as resistance and toxicity persist (Li et al., 2023). Additionally, novel immunotherapies, like immune checkpoint inhibitors, show efficacy in only a limited subset of patients, underscoring the need for more personalized treatment approaches (Cornista et al., 2023; Nikolouzakis et al., 2024).

The tumor microenvironment (TME) is a critical factor in cancer progression, shaping both metastasis and treatment outcomes (Biray Avci et al., 2024). It consists of a diverse array of cells, extracellular matrix components, and signaling molecules that collectively drive tumor growth and resistance to therapies (de Visser and Joyce, 2023). In CRC, transcriptomic analyses have highlighted the TME’s heterogeneity and its influence on prognosis (Sadanandam et al., 2013; De Sousa E Melo et al., 2013; Guinney et al., 2015). Stroma-related gene signatures have been shown to affect CRC’s response to chemoradiotherapy (Gonçalves-Ribeiro et al., 2017). The presence of cytotoxic T cells within the CRC TME has been linked to a more robust immune response against tumor cells (Charoentong et al., 2017). Additionally, features of the TME, such as epithelial-to-mesenchymal transition and angiogenesis, contribute to resistance against immune checkpoint inhibitors (Wang et al., 2018; Hugo et al., 2016). Understanding the complex interactions between cancer cells and the TME is crucial for addressing the challenges of cancer progression and improving therapeutic outcomes.

Metabolic reprogramming in cancer cells, essential for supporting their rapid proliferation and growth, has been well-documented (Nong et al., 2023). Recent studies have underscored how these metabolic shifts also shape the TME by modulating various components within it Chen et al. (2024). In CRC, metabolic alterations significantly impact the levels and activity of immune cells and factors within the TME, influencing patient prognosis (Lu et al., 2021; Bohn et al., 2018; Li et al., 2020; Ji et al., 2021; Yang et al., 2022; Ringel et al., 2020). For instance, changes in glycolysis and lipid metabolism have been linked to the suppression of cytotoxic T cells and the promotion of immunosuppressive cells, such as regulatory T cells and myeloid-derived suppressor cells, which can hinder the anti-tumor immune response (Lu et al., 2021; Bohn et al., 2018; Li et al., 2020; Ji et al., 2021; Yang et al., 2022). Under hypoxic conditions, HIF activation via AKT-mTOR-HIF1α signaling enhances glycolysis in the tumor microenvironment, promoting CRC progression and immune evasion by upregulating glycolytic enzymes and stabilizing HK2, which reduces CD8^+^ T-cell infiltration (Li et al., 2020; Ji et al., 2021). Lipid metabolism supports CRC progression by influencing energy supply, cell proliferation, and immune function, with a high-fat diet impairing CD8^+^ T-cell infiltration and promoting tumor immune evasion (Yang et al., 2022; Ringel et al., 2020). Additionally, the metabolic reprogramming of immune cells during their proliferation, differentiation, and effector function execution is crucial for mounting an effective anti-cancer immune response (Xia et al., 2021). Hence, there is significant crosstalk between metabolic reprogramming and the TME in CRC, influencing disease progression and therapeutic resistance. This study aims to systematically dissect this interplay using transcriptomic data and bioinformatics approaches to characterize CRC heterogeneity and identify potential metabolic and TME-based therapeutic targets. Our approach serves a dual purpose: identifying metabolic factors that sustain TME cell viability and cancer cell-derived metabolic mechanisms that enhance TME infiltration and remodeling.

## Methods and materials

### Identification of TME- and metabolism-related genes

We compiled a comprehensive set of 4,061 tumor microenvironment (TME) genes by integrating data from multiple principal studies (Aran et al., 2017; Newman et al., 2015; Becht et al., 2016; Chifman et al., 2016; Rooney et al., 2015; Tirosh et al., 2016). These genes were curated from three major TME algorithm databases—xCell, CIBERSORT, and MCP-counter (Aran et al., 2017; Newman et al., 2015; Becht et al., 2016). Additionally, immune gene signatures from two large-scale studies spanning multiple cancer datasets (Chifman et al., 2016; Rooney et al., 2015) and TME-specific data from a single-cell melanoma study (Tirosh et al., 2016) were included. For metabolism-related analysis, we extracted 944 genes from the Molecular Signatures Database (MSigDB), focusing on gene signatures linked to KEGG (Kyoto Encyclopedia of Genes and Genomes) pathways relevant to metabolic processes (Supplementary Table S1). We eliminated duplicate genes (n = 211), resulting in a final count of 3,850 genes in the TME dataset.

### Data acquisition and identification of TMET crosstalk genes

Raw count data for colorectal adenocarcinoma (COAD), rectal adenocarcinoma (READ), and normal colon samples from the GTEx project were retrieved using the “TCGArecount2_query” function in the “TCGAbiolinks” R package, which accesses RNA sequencing data from the Recount2 project. Differential expression analysis (DEA) was performed with the “TCGAanalyze_DEA” function, utilizing the “edgeR” pipeline and the generalized linear model likelihood ratio test (glmLRT) method. Genes were considered significantly differentially expressed based on an FDR cutoff of 0.05 and a log2 fold change (logFC) threshold of ±1.

Four independent gene expression datasets (GSE23878, GSE44076, GSE110224, and GSE41258) were obtained from the Gene Expression Omnibus (GEO) database, comprising CRC patient samples (n = 336) and normal controls (n = 198) to cross-validate the outcomes from TCGA-GTEx DEA. Gene expression data from each dataset were normalized using the “normalizeBetweenArrays” function in the “limma” package, with log2 transformation applied where necessary to stabilize variance before averaging replicates. To integrate data across multiple datasets, common genes were identified, and batch effects were corrected using the “ComBat” function from the sva package. Differential gene expression was analyzed using the “limma” package in R by applying logFC >0.585, adj.P.Val <0.05.

### Construction of the training meta-cohort

For consensus clustering, we first constructed a meta-cohort (n = 1,403) comprising TCGA CRC (COAD, READ) and two GEO datasets (GSE17538, GSE39582). Gene expression data were processed using the same normalization and batch correction procedures described above.

### Consensus clustering

The CRC patients from meta-cohort (n = 1,403) were clustered based on the expression data for 257 TMET crosstalk DEGs with significant protein-level interactions using the Non-negative Matrix Factorization (NMF) algorithm (Gaujoux and Seoighe, 2010). Prognostic TMET genes were selected by univariate Cox regression and clustered using NMF, with cluster stability evaluated across k = 2–10 using cophenetic correlation.

### Enrichment pathways analysis

To evaluate the enrichment of TMET crosstalk DEGs, we utilized ShinyGO version 0.80 (http://bioinformatics.sdstate.edu/go/), focusing on KEGG pathways. Additionally, to quantify the enrichment of hallmark cancer pathways and immune cell pathways across the clusters, we employed the “GSVA” package in R. To further investigate the functional roles of DEGs between clusters, Gene Ontology (GO) and KEGG pathway enrichment analyses were conducted using the “clusterProfiler” package for Gene Set Enrichment Analysis (GSEA).

### Copy number variation data acquisition and processing

Level 4 GISTIC2-derived copy number variation (CNV) data for colorectal cancer (COADREAD) were obtained from the Broad Institute’s Firehose GDAC platform (28 January 2016, analysis run) (https://gdac.broadinstitute.org). The dataset, “gdac.broadinstitute.org_COADREAD-TP.CopyNumber_Gistic2.Level_4.2016012800.0.0,” contains thresholded CNV calls for tumor samples, generated using the GISTIC2 algorithm. The focal segment file was stratified by clusters and used as input for GISTIC2.0 (Genomic Identification of Significant Targets in Cancer) via GenePattern (https://cloud.genepattern.org). SNP6 copy number analysis was performed to obtain GISTIC scores, focal amplifications, and deletions for each cluster. The focal and broad CNV data were further analyzed to calculate focal- and arm-level CNV burden across clusters.

### Mutation data acquisition and analysis

Simple Nucleotide Variation data for the TCGA CRC cohort were retrieved from the TCGA Data Portal (https://portal.gdc.cancer.gov/) using the “TCGAbiolinks” R package. Gene mutation analysis and oncoplot visualization were performed using the “maftools” R package.

### Tumor microenvironment characterization

The cellular composition of the tumor microenvironment was estimated using MCP-counter to quantify immune and stromal cell populations, and tumor purity was inferred using the ESTIMATE algorithm (Becht et al., 2016; Yoshihara et al., 2013).

### Single-cell data processing

To investigate TMET gene expression at the single-cell level, we utilized The Tumor Immune Single-cell Hub (TISCH) (http://tisch.comp-genomics.org/), a database providing curated TME annotations (Sun et al., 2021). TISCH employs the MAESTRO pipeline for preprocessing, so additional quality control steps were unnecessary (Wang et al., 2020). We analyzed two colorectal cancer single-cell datasets: EMTAB8107 (7 patients, 23,176 cells) and GSE166555 (12 patients, 66,050 cells). Single-cell data were analyzed using Seurat, with normalization (NormalizeData), feature selection (FindVariableFeatures), dimensionality reduction (RunPCA, RunTSNE), and TMET gene scoring (AddModuleScore) across annotated cell types.

### Risk score calculation using Lasso-Cox regression

To construct a risk score based on two genes, we applied Lasso-Cox regression using the glmnet and survival R packages. A Cox proportional hazards model with Lasso regularization was fitted using glmnet(), and the optimal lambda was determined via cross-validation using cv.glmnet(). The “coef()” function was used to extract nonzero coefficients, identifying the two genes included in the risk model. The risk score was computed for each sample as a weighted sum of gene expression values using crossprod(), and patients were classified into high- and low-risk groups based on the median risk score.

### External validation

To validate the prognostic significance of PDE2A and CKMT2 in independent cohorts, we employed the Kaplan-Meier Plotter database’s CRC domain (https://kmplot.com/). Recurrence-free survival and overall survival were analyzed using data from 1,336 to 1,061 CRC patients, respectively, derived from 15 GEO datasets.

### Immunotherapy response

The immunotherapy domain of the Kaplan-Meier Plotter database was used to assess the impact of PDE2A and CKMT2 on immunotherapy response. This domain includes data on patient responses to three immune checkpoint inhibitors—anti-PD1 (n = 520), anti-PD-L1 (n = 486), and anti-CTLA-4 (n = 121)—across nine cancer types, including glioblastoma, lung cancer, head and neck cancer, melanoma, bladder and urothelial cancer, esophageal cancer, and liver cancer. Additionally, the immunotherapy domain from the ROC Plotter database was employed, offering comparable data on immunotherapy responses, with box plots for treatment response outcomes and ROC plots to assess predictive performance (Kovács et al., 2023).

### Cell lines and cell culture

This study employed the normal human colorectal mucosal cell line FHC alongside two colorectal cancer cell lines, SW480 and HCT116. All cell lines were sourced from the Cell Bank of the Chinese Academy of Sciences (Shanghai, China). Cells were maintained in Dulbecco’s Modified Eagle Medium (DMEM) supplemented with 10% fetal bovine serum (FBS), 100 U/mL penicillin, and 100 μg/mL streptomycin. Cultures were kept at 37 °C in a humidified atmosphere containing 5% CO_2_. Cell identity and contamination status were routinely checked by assessing morphological features and performing *mycoplasma* screening.

### Quantitative real-time PCR

Total RNA was extracted using the EZBioscience RNA Purification Kit (#B0004D). First-strand cDNA synthesis was performed using the HiScript® III RT SuperMix for qPCR (+gDNA wiper) (Vazyme, #R323-01). Quantitative PCR was carried out with ChamQ Universal SYBR qPCR Master Mix (Vazyme, #Q711-02) following the manufacturer’s instructions. Primer sequences used for amplification are provided in Table 1.

**TABLE 1:** Primers used in this study.

Genes	Primers
CKMT2-Forward	CCA​AGC​GCA​GAC​TAC​CCA​G
CKMT2-Reverse	GGT​GTC​ACC​TTG​TTG​CGA​AG
PDE2A-Forward	GAA​AGT​CCG​GGA​GGC​TAT​CAT
PDE2A-Reverse	CAC​TTG​GGT​ATC​AGG​AGC​CA

### Western blotting

Cells were rinsed twice with phosphate-buffered saline (PBS) and lysed in RIPA buffer (Cell Signaling Technology, United States) supplemented with protease and phosphatase inhibitors (CWBIO, China). The lysates were cleared by centrifugation at 12,000 × g for 15 min at 4 °C. Protein concentrations were quantified using the BCA Protein Assay Kit (CWBIO, China). Equal protein amounts (30 µg) were resolved by SDS-PAGE and transferred onto PVDF membranes (Bio-Rad, Minneapolis, MN, United States). Membranes were blocked with 5% bovine serum albumin (BSA) in TBST buffer and incubated overnight at 4 °C with a primary antibody against CKMT2 (Proteintech, #23995-1-AP, Rabbit, 1:1,000) and PDE2A (Proteintech, #55306-1-AP,Rabbit, 1:1,000). After washing, membranes were incubated with an HRP-conjugated anti-rabbit secondary antibody (Beyotime, #A0208, 1:1,000) for 1 h at room temperature. Signals were visualized using an enhanced chemiluminescence detection system (Pierce Biotechnology).

### Clinical specimens

Tumor and adjacent normal tissue samples were collected from 63 colorectal cancer patients between December 2022 and August 2024 at the Department of Radiation Oncology, Shenzhen People’s Hospital (The Second Clinical Medical College, Jinan University; The First Affiliated Hospital, Southern University of Science and Technology), Shenzhen, China. Written informed consent was obtained from all participants, and the study was approved by the Institutional Review and Ethics Board of Shenzhen People’s Hospital (Approval number: LL-KY-2024137-01).

### Immunohistochemical analysis

Immunohistochemistry (IHC) was performed on 4 µm sections of formalin-fixed, paraffin-embedded tumor and adjacent normal tissues obtained from colorectal cancer patients. Sections were deparaffinized using xylene and graded ethanol, followed by antigen retrieval in citrate buffer (pH 6.0) via microwave heating. To block endogenous peroxidase activity, sections were treated with 0.3% hydrogen peroxide and subsequently incubated in 5% bovine serum albumin (BSA) in phosphate-buffered saline (PBS) to minimize nonspecific binding. Primary antibodies, including anti-CKMT2 (Proteintech, #13207-1-AP, rabbit, 1:1,000), anti-PDE2A (Proteintech, #55306-1-AP, rabbit, 1:200), were applied, and sections were incubated overnight at 4 °C. The next day, sections were incubated with biotinylated secondary antibodies at room temperature, and staining was developed using 3,5-diaminobenzidine (DAB) as the chromogen. Hematoxylin was used for counterstaining. Staining intensity was graded on a scale of 0–3 (0: no staining, 1: weak, 2: moderate, 3: strong), while the proportion of positively stained cells was scored from 0 to 4 (0: <5%, 1: 5%–25%, 2: 26%–50%, 3: 51%–75%, 4: >75%). The final IHC score was calculated by multiplying the intensity and frequency scores. For tissues exhibiting heterogeneity, individual regions were scored separately, and the results were combined to produce the final score.

### Statistical analysis

All statistical analyses were performed using R v4.0.3 (http://www.r-project.org). Categorical variables were analyzed using the chi-square test, while continuous variables were compared between two groups using either the Student’s t-test or the Wilcoxon rank-sum test, depending on data distribution. For comparisons across multiple groups, ANOVA or the Kruskal-Wallis test was applied. Survival analysis was conducted using the Kaplan-Meier method, with differences assessed by the log-rank test. Correlations between variables were evaluated using Spearman’s or Pearson’s correlation coefficients.

## Results

### Identification of crosstalk between TME- and metabolism-related factors

**FIGURE 1:**
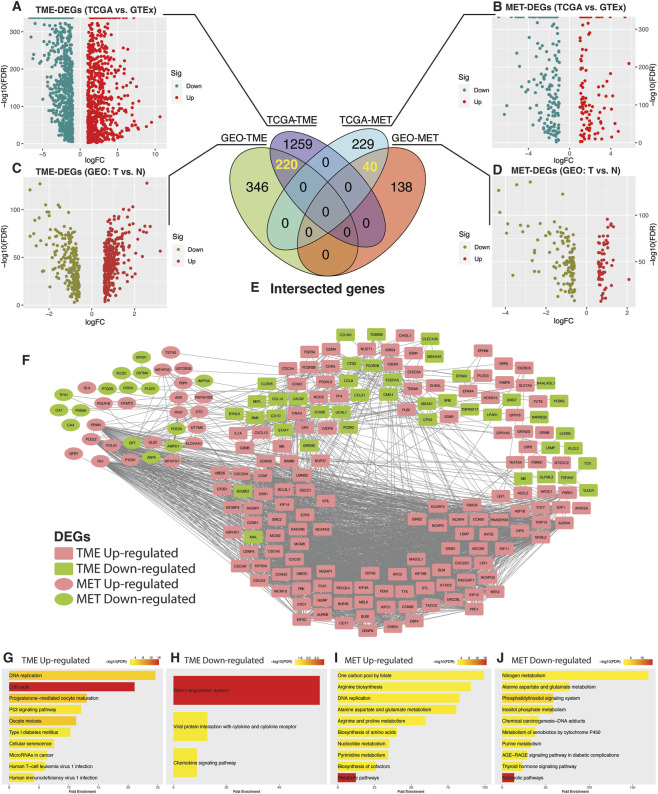
Identification of TMET crosstalk genes. **(A–D)** Volcano plots showing differential expression of tumor microenvironment (TME)–related and metabolism-related (MET) genes in colorectal cancer. Upper panels compare TCGA colorectal tumors with GTEx normal colon tissues (**(A)** TME-DEGs; **(B)** MET-DEGs) using edgeR (|logFC| ≥ 1, FDR ≤0.05). Lower panels compare GEO colorectal tumors with normal tissues (**(C)** TME-DEGs; **(D)** MET-DEGs) using limma (|logFC| ≥ 0.585, FDR ≤0.05). **(E)** Venn diagram showing overlapping TMET crosstalk genes (220 TME-related and 40 MET-related genes). **(F)** Protein-protein interaction (PPI) network illustrating interactions among TMET crosstalk genes. **(G–J)** KEGG pathway enrichment analysis of TMET crosstalk genes performed using ShinyGO (v0.80).

We analyzed the differential expression of tumor microenvironment (TME)- and metabolism (MET)-related genes in colorectal cancer (CRC) to assess their significance in tumorigenesis. First, we compared the mRNA expression levels of these genes between CRC samples (n = 669) from TCGA and normal colon tissues from GTEx (n = 376). Differential analysis yielded 1,479 differentially expressed TME-related genes and 269 metabolism related genes (Figures 1A,B). To further assess the robustness of these findings, we performed cross-validation using a meta-cohort comprising four GEO datasets (GSE23878; GSE44076; GSE110224; GSE41258), that included both CRC (n = 336) and normal colon samples (n = 198). Differential analysis in this cohort identified 566 and 178 TME- and MET-related DEGs, respectively (Figures 1C,D). Intersection of the TCGA-GTEx and GEO analyses revealed 220 and 40 unique TME- and MET-related DEGs shared across datasets (Figure 1E). The Protein-protein interaction (PPI) network demonstrated significant interactions between TME- and MET-related DEGs (Figure 1F). TME upregulated genes were enriched in DNA replication and cell cycle pathways while downregulated genes were involved in chemokine signaling (Figures 1G,H). In parallel, both upregulated and downregulated MET-related genes were enriched in pathways supporting metabolic activity, including nucleotide and pyrimidine metabolism and several pathways associated with amino acid metabolism (Figures 1I,J). These intersecting genes, hereafter referred to as TMET genes, were selected for subsequent downstream analyses.

### TMET crosstalk-based subtyping of CRC

**FIGURE 2:**
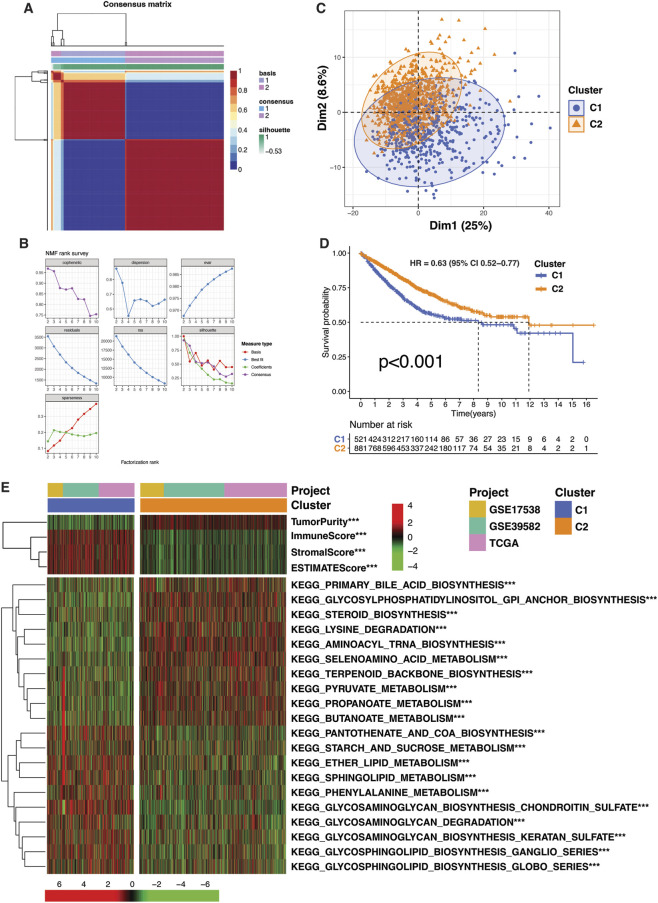
TMET crosstalk-based molecular subtypes of colorectal cancer. **(A)** Cophenetic correlation coefficients and **(B)** consensus clustering heatmap derived from non-negative matrix factorization (NMF), supporting k = 2 as the optimal clustering solution. **(C)** Principal component analysis (PCA) demonstrating separation of TMET subtypes based on TMET gene expression. **(D)** Kaplan–Meier overall survival curves comparing TMET subtypes (C1: high-risk; C2: low-risk). **(E)** Heatmap summarizing ESTIMATE scores and KEGG metabolic pathway enrichment across TMET subtypes. Statistical significance was assessed using the Wilcoxon test (*P < 0.05; **P < 0.01; ***P < 0.001).

To explore the clinical and functional relevance of the TMET crosstalk genes in CRC, we performed consensus clustering based on their expression profiles. A meta-cohort of CRC samples was created by integrating transcriptomic data from TCGA CRC samples (including colon and rectal adenocarcinoma [COAD and READ]) and the GSE17538, and GSE39582 datasets. To enhance data comparability and reproducibility, batch effects were corrected. The expression profiles of 260 TMET crosstalk genes was then used for molecular subtype identification through non-negative matrix factorization (NMF) consensus clustering. Two optimal clusters (k = 2) were identified, based on the visual inspection of the consensus matrix and cophenetic correlation coefficients (Figures 2A,B). To validate the clustering results, principal component analysis (PCA) was performed using the expression data of the 260 TMET genes. The PCA plot effectively visualized cluster separation, with Dim1 (PC1) capturing 25% of the total variation, confirming the robustness of the identified subtypes (Figure 2C). Moreover, a significant difference in survival outcomes was observed between the two clusters, with C1 exhibiting the poorest prognosis (p < 0.001) (Figure 2D). Additionally, we visualized the clusters in the context of TME by utilizing the ESTIMATE algorithm. The cluster 1 was enriched in TME components, such as immune and stromal components (Figure 2E). Functional enrichment analysis of the KEGG pathways showed C1 to be more involved in energy production, extracellular matrix remodeling, and lipid metabolism (through sphingolipids, glycosphingolipids, and fatty acids). These features align with characteristics of a highly aggressive cancer phenotype, where cells rely heavily on glucose metabolism (Warburg effect), ECM modification for invasion, and energy from lipid metabolism. In contrast, C2 showed upregulation of pathways related to steroid biosynthesis, amino acid metabolism, and membrane dynamics (via GPI-anchor biosynthesis). These metabolic differences suggest that TMET Cluster 1 (hereafter referred to as the C1 subtype) could represent a more aggressive and invasive, lipid metabolism-driven phenotype, whereas TMET Cluster 2 (C2 subtype) may exhibit a prolfierative subtype with unique regulatory mechanisms in steroid and amino acid metabolism.

### Clinical features of TMET CRC subtypes

**FIGURE 3:**
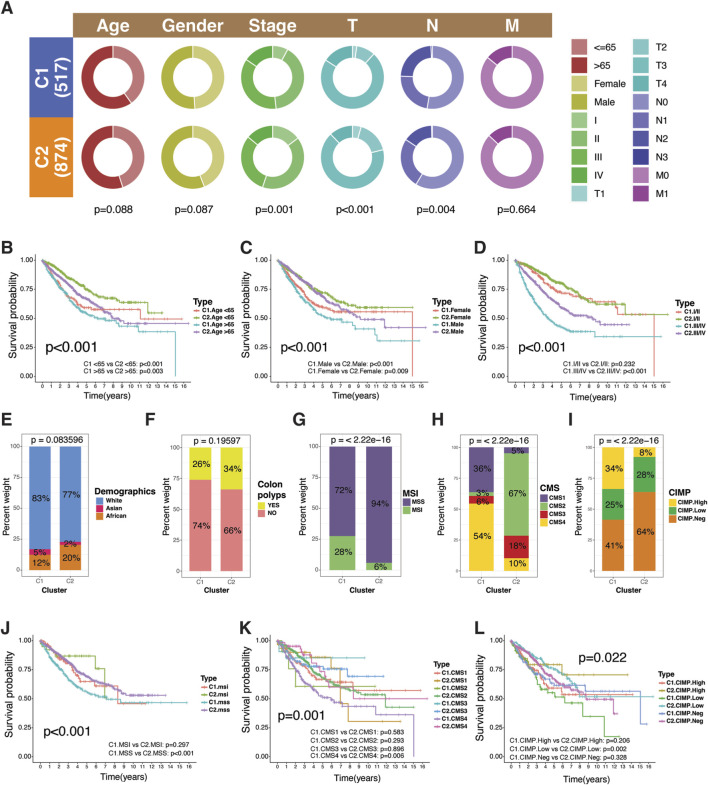
Clinical and molecular characteristics of TMET subtypes. **(A)** Circular plot showing the distribution of age, sex, and tumor stage across TMET subtypes. **(B–D)** Kaplan–Meier survival analyses stratified by TMET subtype and clinical variables (age, sex, stage). **(E–I)** Distribution of TMET subtypes across demographic features, colon polyp status, and established CRC molecular classifications (MSI, CMS, CIMP). **(J–L)** Kaplan–Meier survival curves evaluating the prognostic impact of TMET subtypes within MSI, CMS, and CIMP subgroups. C1: high-risk; C2: low-risk.

C1 subtype had a slightly higher proportion of participants were over 65 years old and male (Figure 3A). There was a significant difference between the TMET subtypes in terms of cancer stage, particularly in the T (tumor size) and N (nodal status) parameters. More participants in Stage I/II disease (including T1/T2 and N0/N1) were found in the C2 subtype compared to the C1 subtype (Figure 3A). C1 exhibited significantly higher nodal involvement compared to C2, aligning with its more invasive phenotype. However, no significant difference was observed between the clusters in terms of distant metastasis. This may be attributed to the relatively small number of patients with available distant metastasis data—only 146 out of approximately 1,400—limiting the statistical power to detect differences in this outcome.

The prognostic differences between the TMET subtypes remained consistent across age and gender subgroups (Figures 3B,C). Notably, no survival differences were observed between the TMET subtypes for patients in Stage I/II (Figure 3D). In other words, being in a particular subtype may not affect the survival outcome for early-stage patients as much as it does for those in more advanced stages. There was no difference between the TMET subtypes for demographics, presence of polyps (Figures 3E,F).

The relationship of TMET subtypes with other classification of CRC was sought. Microsatellite instability was predominant in C1 (Figure 3G). As such, C1 was dominated by the CMS1 (MSI Immune) and CMS4 (Mesenchymal) subtypes of the Consensus Molecular Subtypes (CMS) of CRC while C2 had predominant CMS2 (Canonical) and CMS3 (Metabolic) subtypes (Figure 3H). The C1 had the presence of CpG island methylator phenotype (CIMP)-High subtype (34%) while C2 had predominance of CIMP negative subtype (64%) (Figure 3I). These results highlight C1 as a distinct subgroup characterized by strong epigenetic regulation, immune activity, and mesenchymal features. Prognostic analysis revealed a survival difference between the clusters for MSS (p<0.001), CMS4 (p = 0.006) and CIMP-Low (p = 0.002) subgroups (Figures 3J–L). The lack of prognostic impact in other subtypes could probably be attributed to the extreme imbalance of participants in one of the comparative clusters.

### Mutational landscape of the TMET CRC subtypes

**FIGURE 4:**
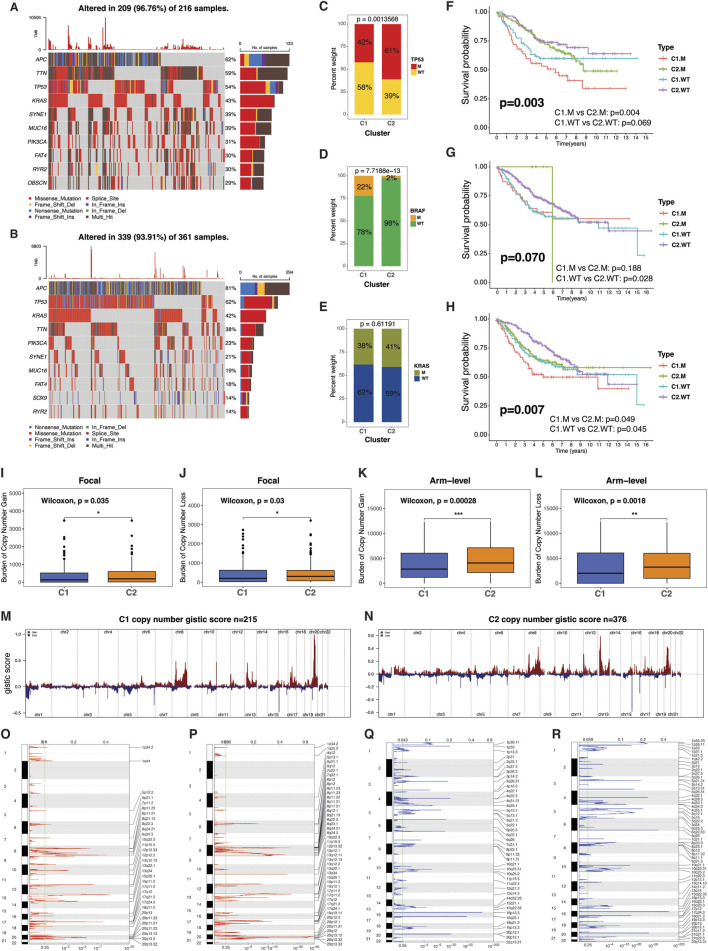
Mutational alterations associated with TMET subtypes. **(A,B)** Oncoplots showing the mutation landscape of the most frequently altered genes in TMET subtypes. **(C–E)** Distribution of TMET subtypes according to TP53, BRAF, and KRAS mutation status. **(F–H)** Kaplan–Meier survival analyses integrating TMET subtype with TP53, BRAF, and KRAS mutations. **(I–L)** Comparison of focal- and arm-level copy number alteration (CNA) burdens between TMET subtypes. **(M,N)** Genome-wide CNA frequency plots (amplifications in red; deletions in blue). **(O–R)** GISTIC 2.0 analysis highlighting significant focal amplification and deletion peaks in TMET C1 and C2 subtypes. C1: high-risk; C2: low-risk.

We conducted an in-depth analysis of genomic aberrations across the TMET CRC subtypes to uncover mutational patterns. The oncoplot visualizations illustrate distinct mutation distributions between TMET subtypes (Figures 4A,B). In the TCGA CRC cohort, C1 exhibited a higher mutation frequency in TTN, along with less frequently mutated genes, including PIK3CA, SYNE1, MUC16, FAT4, and RYR2 (Figure 4A). Conversely, APC and TP53 mutations were significantly more frequent in the C2 subtype, suggesting association with this group (Figure 4B). Additionally, data from the GEO dataset (GSE39582) corroborated these findings, showing a predominance of TP53 mutations in C2, while mutations in KRAS were evenly distributed across TMET subtypes (Figures 4C,D). Notably, BRAF mutations were more frequent in C1 compared to the C2 subtype (Figure 4E). The plateau followed by a sharp decline in the C2.M survival curve likely reflects the small subgroup size (n = 8) and low event rate, with only one recorded death and the remaining patients censored. The mutation status of TP53 significantly influenced the prognostic impact of TMET subtype classification, while the prognostic significance of the clusters persisted regardless of KRAS mutation status (Figures 4F–H).

Copy number alterations (CNAs) have been linked to immune cell infiltration and the response to immune checkpoint blockade therapy (Roh et al., 2017). Our analysis of copy number variation between TMET subtypes revealed that C1 exhibited a significantly lower burden of both focal- and arm-level gains and losses compared to the C2 subtype (Figures 4I–L). This suggests a more stable genomic profile in C1. The GISTIC score, which quantifies the amplitude and frequency of aberrations, further illustrated that amplifications and deletions were markedly more frequent in C2, with notable increases in the number and intensity of these genomic alterations (Figures 4M–R). Figures 4M,N depict GISTIC scores for individual samples, and Figures 4O–R highlight the substantial differences between the TMET subtypes, particularly the more abundant peaks of amplifications and deletions in C2, reflecting its higher genomic instability. These results suggest a link between CNAs and tumor heterogeneity, which may influence the immune landscape and therapeutic outcomes in colorectal cancer.

These findings underscore the molecular diversity between the TMET subtypes, with C2 showing a relatively classic tumor suppressor profile, while C1 presents a broader range of mutations linked to mesenchymal and immune features.

### Transcriptomic differences between TMET subtypes

**FIGURE 5:**
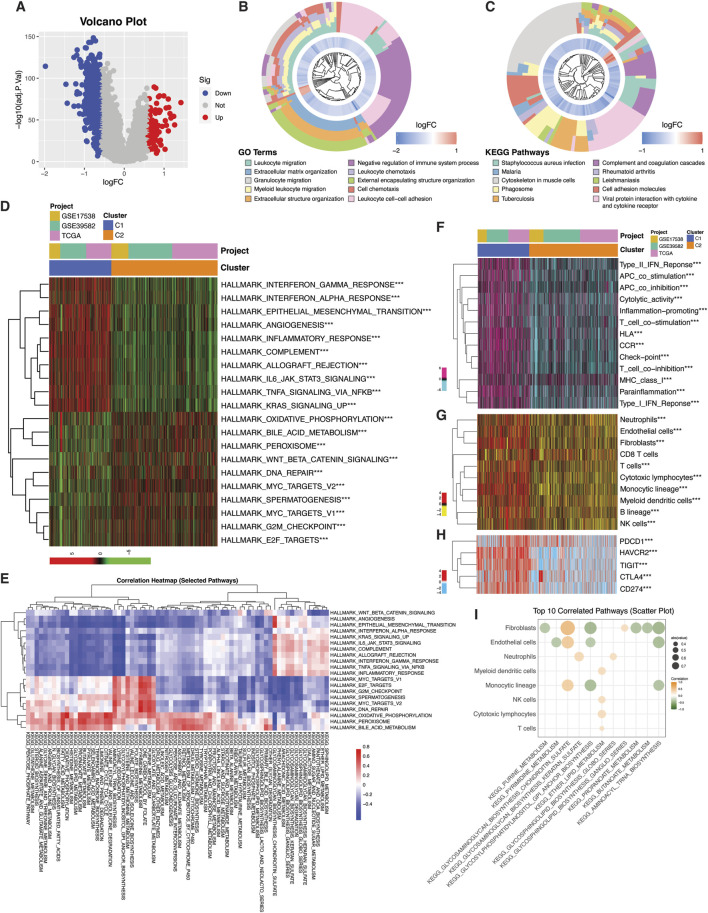
Transcriptomic and pathway differences between TMET subtypes. **(A)** Volcano plot of differentially expressed genes (DEGs) between TMET subtypes. **(B,C)** GO and KEGG pathway enrichment analyses of subtype-specific DEGs. **(D)** ssGSEA heatmap showing differential enrichment of Cancer Hallmark pathways between TMET subtypes. **(E)** Correlation heatmap between KEGG metabolic pathways and Cancer Hallmark pathways. **(F)** ssGSEA-based enrichment of immune-related pathways across TMET subtypes. **(G)** MCP-counter–based estimation of immune and stromal cell infiltration. **(H)** Differential expression of immune checkpoint–related genes. **(I)** Bubble plot illustrating correlations between immune/stromal cell populations and metabolic pathways.

To investigate the functional differences between TMET subtypes, we conducted differential expression analysis (Figure 5A). A total of 565 differentially expressed genes (DEGs) (79 upregulated, 486 downregulated) with a logFC ≥0.5 and an adjusted p-value <0.05 were identified. These DEGs were enriched in biological processes related to immune system regulation, inflammation, infection and immune response, as well as extracellular matrix remodeling and cell migration, as determined by Gene Ontology (GO) and KEGG pathway analysis (Figures 5B,C). The involvement of immune activity was further shown by the enrichment of hallmark immune-related pathways in the C1 subtype, along with enrichment of epithelial-mesenchymal transition (EMT) and angiogenesis (Figure 5D). The C2 subtype exhibited enrichment in gene sets associated with DNA repair and cell cycle related pathways critical for tumor proliferation, with an enhanced metabolic dependence on oxidative phosphorylation. The heatmap highlights the correlation between the hallmark pathways and KEGG metabolic pathways differentially active between the TMET subtypes (Figure 5E). For example, lipid metabolism (glycosaminoglycan, glycosphingolipids, and fatty acids) is positively correlated with immune and inflammation-related hallmark pathways in C1. Delineation of immune response pathways indicated the activation of all 13 pathways related to inflammation, chemokine receptors (CCR), Type I/II interferon responses, antigen presentation (MHC class I, HLA), T cell activation and inhibition, as well as checkpoints commonly linked to T cell exhaustion (Figure 5F). MCP counter results showed enhanced enrichment of all the immune and stromal cells except CD8 T cell (Figure 5G). Nonetheless, CD8 T cell exhaustion markers, also known as immune inhibitory checkpoints, were expressed abundantly in C1, indicating a possible difference in the phenotype of CD8 T cells (naïve and exhausted) (Figure 5H). Moreover, metabolic pathways linked to the C1 subtype, including glycosaminoglycan and glycosphingolipid metabolism, showed a positive correlation with fibroblasts and endothelial cells, whereas those associated with the C2 subtype exhibited a negative correlation with these cell types (Figure 5I).

### Individual prognostic and functional assessment of TMET genes

**FIGURE 6:**
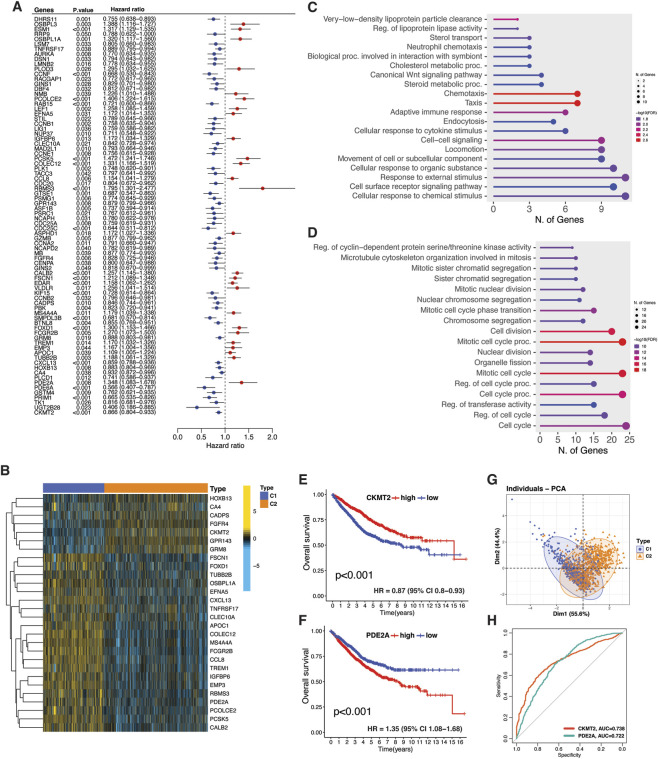
Prognostic TMET genes defining TMET subtype phenotypes. **(A)** Univariate Cox regression analysis of TMET crosstalk genes in CRC. **(B)** Heatmap of the top 28 prognostically relevant TMET genes with significant differential expression (|logFC| > 0.15). **(C,D)** KEGG pathway enrichment of C1- and C2-upregulated TMET genes. **(E,F)** Kaplan–Meier survival curves stratified by CKMT2 and PDE2A expression. **(G)** PCA plot showing subtype separation driven by CKMT2 and PDE2A expression. **(H)** ROC curves evaluating the discriminatory performance of CKMT2 and PDE2A.

We analyzed the prognostic significance and functional implications of 260 TMET genes in CRC. Out of these, 80 genes were significantly associated with CRC prognosis, 54 were associated with favorable survival (hazard ratio <1) and 26 were associated with unfavorable survival (hazard ratio >1) (Figure 6A). Among these, 72 prognostic genes exhibited significant differential expression between clusters. The heatmap highlights 28 genes with the most significant log fold changes (logFC >0.15) (Figure 6B). GO analysis revealed that the upregulated genes were predominantly linked to biological processes such as chemotaxis, immune response, and lipid metabolism, while downregulated genes were associated with cell cycle regulation and cell division (Figures 6C,D). This suggests that the 72 differentially expressed genes play a pivotal role in functional and prognostic impact of TMET subtype classification in CRC.

Consistent with the distinct tumor-intrinsic and stromal-immune metabolic programs previously identified between TMET subtypes (Figure 5), we next examined the prognostic relevance of individual metabolic genes. Specifically focusing on metabolic components, two key metabolic genes, PDE2A (C1 subtype) and CKMT2 (C2 subtype), demonstrated opposite prognostic trends in CRC patients (Figures 6E,F). PCA showed that the expression of these two genes accounted for 55.6% of the variation between clusters on PC1 and 44.4% on PC2, suggesting their substantial role in defining cluster distinctions (Figure 6G). Furthermore, ROC analysis demonstrated that both genes had an AUC exceeding 70, indicating their strong predictive power in distinguishing between clusters (Figure 6H). To clarify the cellular origins underlying these divergent prognostic effects, we next examined TMET gene expression at single-cell resolution.

### TMET factors at single-cell resolution

**FIGURE 7:**
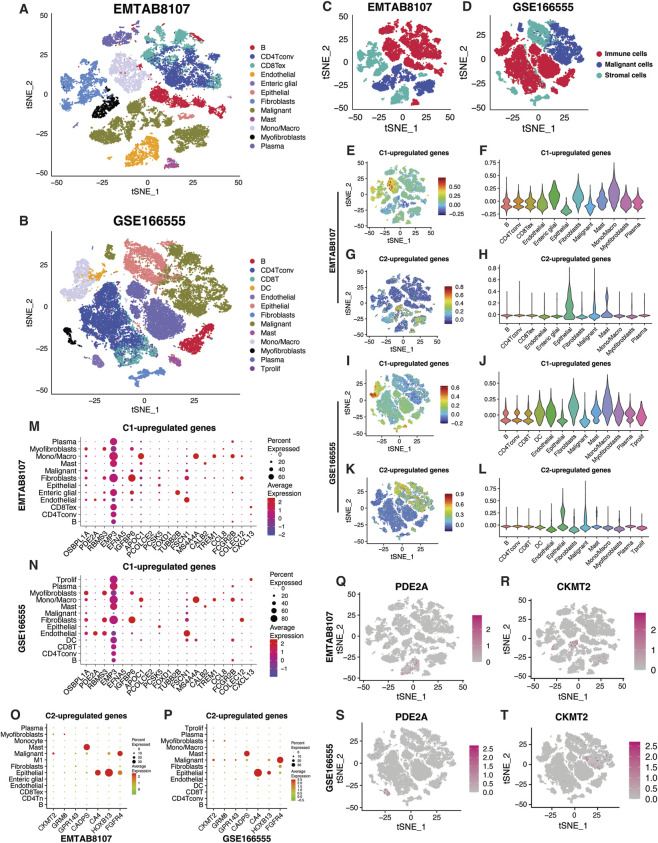
Single-cell resolution of TMET gene expression. **(A,B)** t-SNE plots showing major cell populations in CRC single-cell datasets EMTAB8107 and GSE166555. **(C,D)** Distribution of malignant, immune, and stromal lineages across datasets. **(E–H)** Expression patterns of C1- and C2-associated TMET genes in EMTAB8107. **(I–L)** Corresponding expression patterns in GSE166555. **(M–P)** Dot plots showing TMET gene expression across cell types. **(Q–T)** t-SNE plots illustrating PDE2A and CKMT2 expression in both datasets. C1: high-risk; C2: low-risk.

Our previous analysis indicated a higher abundance of immune and stromal cells within the TME of the C1 subtype. To further validate this observation at a single-cell resolution, we analyzed two CRC single-cell RNA sequencing datasets: EMTAB8107 (7 patients, 23,176 cells) and GSE166555 (12 patients, 66,050 cells) (Figures 7A,B). These datasets encompass the three major components of the TME: tumor, immune, and stromal cells (Figures 7C,D). Focusing on the 28 significantly differentially expressed TMET genes, we observed distinct expression patterns across the TME (Figures 7E–L). TMET genes linked to the C1 subtype were upregulated in endothelial cells, fibroblasts, and monocytes/macrophages (Mono/Mac), indicating a stronger stromal and immune cell presence (Figures 7E,F). Conversely, TMET genes associated with the C2 subtype were predominantly expressed in epithelial and malignant cells (Figures 7G,H). These observations were also validated in the GSE166555 dataset (Figures 7I–L). Dot plots indicate the expression levels of individual TMET genes in each cell type across both CRC datasets (Figures 7M–P). Notably, CKMT2 showed selective expression in subsets of malignant cells, while PDE2A was predominantly expressed in endothelial cells, reinforcing the metabolic heterogeneity between TMET subtypes (Figures 7M–T). This single-cell level validation highlights the distinct cellular compartmentalization of TMET genes across subtypes, with C1 marked by stromal–immune enrichment and C2 by tumor-centric metabolic gene expression, providing cellular context for the subtype-specific transcriptomic patterns observed at the bulk transcriptomic level.

### CRC risk stratification based on PDE2A and CKMT2

**FIGURE 8:**
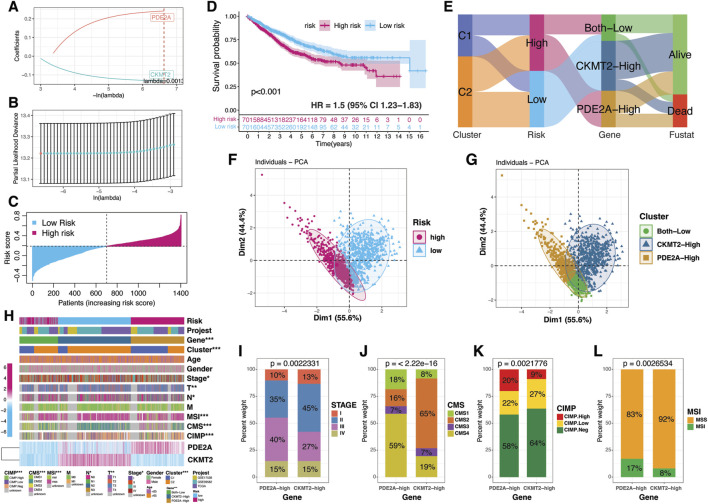
Risk stratification based on PDE2A and CKMT2. **(A,B)** Risk stratification of CRC patients using LASSO regression based on PDE2A and CKMT2 expression. **(C)** Bar plot illustrating the distribution of CRC patients according to risk scores derived from PDE2A and CKMT2. **(D)** Kaplan-Meier survival curve comparing overall survival (OS) between risk subgroups. **(E)** Sankey diagram mapping the distribution of CRC patients across molecular clusters, risk subgroups, PDE2A and CKMT2 expression levels, and vital status. **(F)** PCA plot visualizing the dimensional separation of risk subgroups. **(G)** PCA plot depicting the segregation of gene-based subgroups. **(H)** Heatmap displaying PDE2A and CKMT2 expression across risk subgroups and their associations with clinical and molecular features. **(I–L)** Bar plots showing the distribution of gene-based subtypes across cancer stages and molecular classifications, including MSI, CMS, and CIMP status.

To investigate the prognostic significance of PDE2A and CKMT2, we developed a risk stratification model based on their expression levels. Using a LASSO-Cox regression model, we derived risk coefficients for each gene, allowing us to compute a risk score for each patient (Figures 8A,B). This score was calculated by summing the mRNA expression values of PDE2A and CKMT2, weighted by their respective Lasso-derived coefficients. The risk distribution plot visualizes the risk scores across the cohort, highlighting a clear distinction between high- and low-risk patients (Figure 8C). Kaplan-Meier survival analysis further demonstrates that patients with higher risk scores exhibit significantly worse survival outcomes (Figure 8D). Sankey diagram visualizes the relationship between clusters, risk subgroups, and the expression patterns of PDE2A and CKMT2 (Figure 8E). Gene-based subgroups were defined using the median expression levels of PDE2A and CKMT2. Notably, the PDE2A-high subgroup was primarily associated with the high-risk group, while CKMT2-high patients were completely derived from the low-risk group. PCA further validated the distinct separation between risk-based and gene-based subgroups (Figures 8F,G).

Clinical feature analysis revealed significant differences between risk subgroups across multiple clinical parameters, including tumor stage, MSI, CMS, and CIMP status (Figure 8H). PDE2A expression was elevated in patients with stage III disease, CMS4, CIMP-high, and MSI status (Figures 8I–L). In contrast, CKMT2 expression was higher in stage II, CMS2, CIMP-negative, and MSS tumors (Figures 8I–L). These findings suggest that PDE2A and CKMT2 are not only key regulators of CRC progression but also markers associated with distinct CRC subtypes, further reinforcing their potential as biomarkers for risk stratification and clinical decision-making.

### Experimental validation of CKMT2 and PDE2A in CRC

**FIGURE 9:**
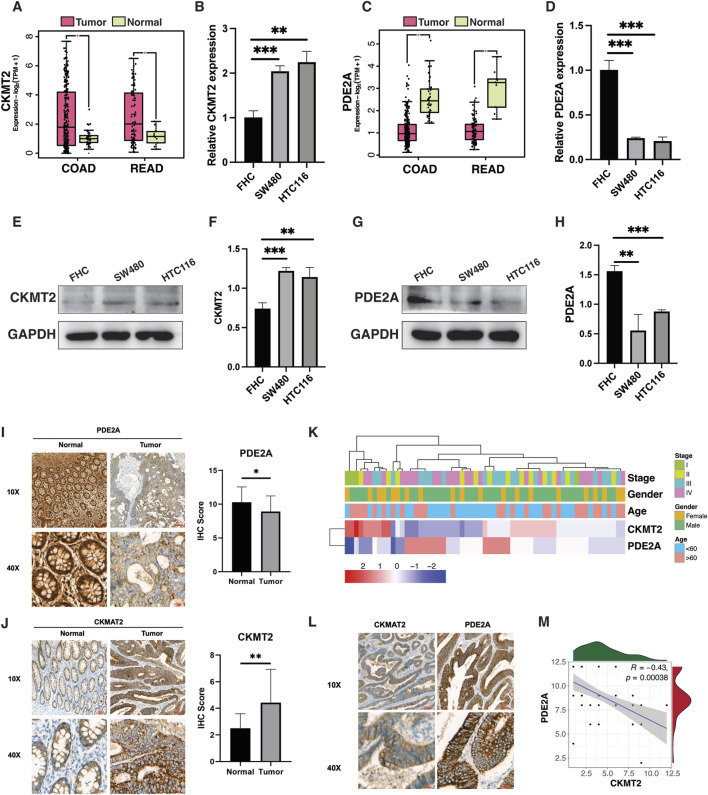
Validation of PDE2A and CKMT2 in colorectal cancer. **(A,C)** GEPIA-based comparison of CKMT2 and PDE2A mRNA expression in normal and CRC tissues. **(B,D)** qPCR validation in FHC, SW480, and HCT116 cell lines. **(E–H)** Western blot analysis and quantification of CKMT2 and PDE2A protein expression. **(I,J)** Representative IHC images and quantitative comparison in CRC and adjacent normal tissues (n = 63). **(K)** Heatmap of protein expression in relation to clinicopathological features. **(L,M)** Representative IHC images and Pearson correlation analysis showing inverse expression of CKMT2 and PDE2A.

To validate these candidate biomarkers, we first assessed CKMT2 mRNA expression in colorectal cancer samples using the GEPIA database. CKMT2 expression was significantly higher in COAD (n = 275) and READ (n = 92) tumor samples compared to normal tissues (COAD: n = 41; READ: n = 10) (Figure 9A). Consistent with these findings, *in vitro* analysis showed that CKMT2 mRNA levels were markedly elevated in colon cancer cell lines (SW480 and HCT116) compared to normal colon cells (FHC) (Figure 9B). In contrast, PDE2A expression was significantly lower in COAD and READ tumor tissues than in normal tissues (Figure 9C), and PCR analysis confirmed its downregulation in SW480 and HCT116 cell lines relative to FHC cells (Figure 9D). Western blot analysis further validated these findings, demonstrating a significant increase in CKMT2 protein expression and a decrease in PDE2A protein expression in cancer cells (p < 0.001) (Figures 9E–H).

Clinical specimens from CRC patients and adjacent normal tissues (n = 63) were analyzed using IHC to assess CKMT2 and PDE2A protein expression. PDE2A was broadly expressed in both CRC and adjacent normal colon tissues but was slightly higher in normal tissues than in tumors, whereas CKMT2 showed significantly higher expression in CRC tissues compared to adjacent normal tissues (Figures 9I,J). These findings align with TCGA RNA-seq data, which revealed elevated CKMT2 and reduced PDE2A expression in tumors relative to normal tissues. Figure 9K illustrates the clinical features and expression patterns of CKMT2 and PDE2A across CRC samples, revealing a visually apparent negative correlation. Representative IHC images highlight the contrasting expression of CKMT2 and PDE2A, with a significant negative correlation further demonstrated in the correlation plot (Figures 9L,M).

### External validation

**FIGURE 10:**
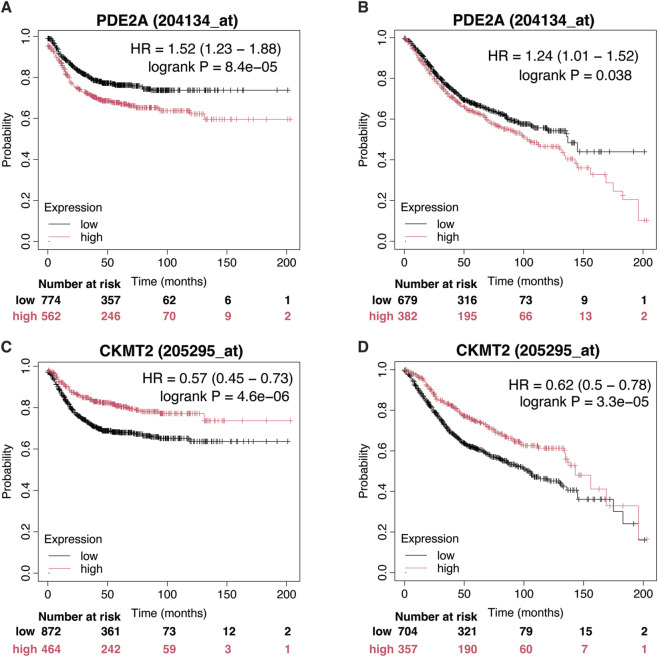
External validation of the prognostic impact of PDE2A and CKMT2. **(A,B)** Kaplan-Meier survival curves illustrating differences in recurrence-free survival (RFS, n = 1,336) and overall survival (OS, n = 1,061) between CRC patients with high and low PDE2A expression. **(C,D)** Kaplan-Meier survival curves depicting differences in recurrence-free survival (RFS, n = 1,336) and overall survival (OS, n = 1,061) between CRC patients with high and low CKMT2 expression.

To validate the prognostic significance of PDE2A and CKMT2 in independent cohorts, we leveraged the Kaplan-Meier Plotter database for CRC (https://kmplot.com/). Survival analysis of recurrence-free survival (RFS, n = 1,336) and overall survival (OS, n = 1,061) consistently demonstrated the opposing prognostic roles of these genes. High PDE2A expression correlated with poorer outcomes, as indicated by worse RFS (HR = 1.52 [1.23–1.88], p < 0.0001) and OS (HR = 1.24 [1.01–1.52], p = 0.038), underscoring its potential as a marker of poor prognosis (Figures 10A,B). Conversely, elevated CKMT2 expression was significantly associated with improved survival, reinforcing its association with improved clinical outcome in CRC (RFS: HR = 0.57 [0.45–0.73], p < 0.0001; OS: HR = 0.62 [0.50–0.78], p < 0.0001) (Figures 10C,D).

### Immunotherapy response

**FIGURE 11:**
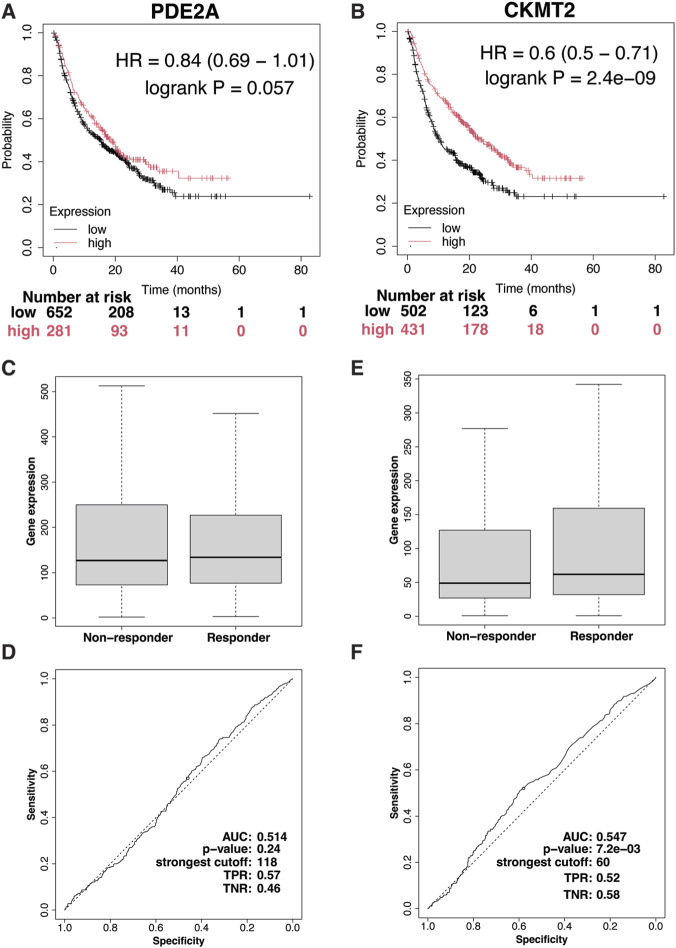
Impact of PDE2A and CKMT2 on immunotherapy response. **(A,B)** Kaplan-Meier survival curves showing overall survival (OS, n = 1,061) differences between patients with high and low PDE2A and CKMT2 expression. **(C,D)** Box plot and ROC curves comparing PDE2A expression between immunotherapy responders (n = 355) and non-responders (n = 570). **(E,F)** Box plot and ROC curves comparing CKMT2 expression between immunotherapy responders (n = 355) and non-responders (n = 570).

The immunotherapy domain of the Kaplan-Meier Plotter database was used to assess the impact of PDE2A and CKMT2 on immunotherapy response. This domain includes data on patient responses to three immune checkpoint inhibitors—anti-PD1 (n = 520), anti-PD-L1 (n = 486), and anti-CTLA-4 (n = 121)—across nine cancer types, including glioblastoma, lung cancer, head and neck cancer, melanoma, bladder and urothelial cancer, esophageal cancer, and liver cancer. PDE2A showed no significant association with immunotherapy response, whereas higher CKMT2 expression was significantly associated with response in this pan-cancer cohort (Figures 11A,B).

To further validate these findings, the immunotherapy dataset from the ROC Plotter database was analyzed, providing comparable response data. PDE2A demonstrated no significant predictive value, with an AUC of 0.514 and a p-value of 0.24 (Figures 11C,D). In contrast, immunotherapy responders (N = 355) exhibited significantly higher pretreatment CKMT2 expression compared to non-responders (N = 570) (Figures 11E,F). ROC analysis evaluating the association between CKMT2 expression and immunotherapy response yielded a statistically significant p-value of 0.0072, though its predictive accuracy remained limited, as indicated by an AUC of 0.547 (Figure 11F). Given that these immunotherapy response analyses were derived from pan-cancer datasets, the findings should be interpreted as exploratory and warrant validation in colorectal cancer–specific cohorts.

## Discussion

We identified 220 TME-related and 40 metabolism-related DEGs in CRC and used these TMET genes to define two biologically and clinically distinct subtypes. TMET C1 was associated with poorer survival and enrichment of immune–mesenchymal and stromal programs, whereas TMET C2 demonstrated features consistent with tumor-intrinsic proliferative activity and greater genomic instability. Metabolically, C1 showed lipid-associated and extracellular remodeling signatures, while C2 was enriched in nucleotide and amino acid metabolism linked to cell cycle processes. Single-cell RNA sequencing further localized C1-associated genes to immune and stromal compartments and C2-associated genes to malignant epithelial cells. Within this framework, PDE2A and CKMT2 emerged as markers of microenvironment-associated and tumor-intrinsic metabolic states, respectively.

Established molecular classification systems—including MSI, CIN, CMS, and CIMP—describe dominant genomic and transcriptional states in CRC (Guinney et al., 2015; Worthley and Leggett, 2010). While these frameworks have improved biological understanding and, in some cases, correlate with prognosis, they were not designed to resolve outcome heterogeneity within individual subtypes or to emphasize prognostic discrimination as a defining criterion (Guinney et al., 2015; Ribic et al., 2003). In contrast, the TMET classification emphasizes prognostic stratification by integrating metabolic programs with their tumor microenvironmental and cellular context, thereby identifying clinically distinct subgroups within established molecular categories.

Colorectal carcinogenesis is commonly described by genomic instability pathways including MSI, CIN, and CIMP (Worthley and Leggett, 2010). Although MSI-high (MSI-H) tumors are generally associated with favorable prognosis and immunotherapy responsiveness, TMET classification revealed outcome heterogeneity within MSI cases, with C1 tumors demonstrating poorer survival (Ribic et al., 2003; Bertagnolli et al., 2009). In TMET C1, stromal enrichment characterized by fibroblast and extracellular matrix signatures, together with TGF-β–associated signaling, aligns with immune exclusion and reduced responsiveness to immune checkpoint blockade (Wang et al., 2018; Hugo et al., 2016). Concurrent lipid-related metabolic activity and stromal-myeloid crosstalk suggest lactate accumulation and altered metabolite signaling, collectively associated with immune exhaustion and limited therapeutic efficacy, providing a biological framework for the adverse outcomes observed in this subgroup (Lu et al., 2021; Bohn et al., 2018; Li et al., 2020; Ji et al., 2021; Yang et al., 2022; Ringel et al., 2020).

The majority of CRCs arise through the chromosomal instability (CIN) pathway, frequently associated with MSS tumors (Worthley and Leggett, 2010). Consistent with this framework, APC and TP53 mutations, hallmarks of CIN-driven tumorigenesis, were more frequent in the TMET C2 subtype (Pino and Chung, 2010; Powell et al., 1992; Menendez et al., 2009). Whereas C1 tumors were enriched for TTN mutations, which have been linked to higher mutational burden and adverse outcomes (Linke, 2018; Wei et al., 2023; Zhao et al., 2023; Oh et al., 2020). These differences indicate that TMET subtypes reflect distinct mutational contexts that intersect with metabolic and microenvironmental states, contributing to prognostic heterogeneity beyond genomic instability alone.

TMET classification further refined prognostic stratification within established transcriptomic and epigenetic frameworks. In advanced-stage disease (III–IV), where outcome heterogeneity remains substantial, TMET distinguished clinically divergent subgroups within CMS categories, particularly separating high-risk CMS4 tumors into biologically and prognostically distinct subsets (Guinney et al., 2015). Similarly, within CIMP-positive CRC, TMET identified subgroups with differential survival, including poorer outcomes among CIMP-low tumors in C1 (Mojarad et al., 2013). These findings reinforce the ability of TMET to resolve clinically relevant heterogeneity within existing molecular classifications.

Single-cell transcriptomic analyses provided functional context for TMET subtypes by linking metabolic gene expression to specific cellular compartments within the tumor microenvironment. In TMET C2, predominant CKMT2 expression in malignant epithelial cells supports a tumor-intrinsic metabolic program characterized by oxidative phosphorylation and proliferative activity, consistent with bulk enrichment of cell cycle and DNA repair pathways. In contrast, TMET C1 displayed elevated PDE2A expression within endothelial and stromal compartments, aligning with stromal dominance and immune-regulatory metabolic remodeling. These compartment-specific patterns reinforce the functional distinction between tumor-intrinsic (C2) and microenvironment-driven (C1) metabolic programs and provide a mechanistic basis for their divergent clinical behavior. In this context, the prognostic associations of CKMT2 and PDE2A should not be interpreted as reflecting simple tumor-suppressive versus oncogenic roles. Rather, their clinical impact appears to arise from the distinct cellular compartments and metabolic contexts in which they are expressed. Tumor-intrinsic metabolic dependencies and microenvironment-mediated metabolic remodeling may exert opposing influences on outcome, despite both contributing to tumor progression.

CKMT2 encodes a mitochondrial creatine kinase involved in ATP buffering and oxidative phosphorylation (Yan, 2016). As part of the phosphocreatine–creatine kinase shuttle system, it contributes to cellular energy homeostasis by facilitating reversible phosphate transfer between ATP and phosphocreatine. Beyond its metabolic role, CKMT2 has been implicated in tumor progression in several malignancies, including osteosarcoma and gastric cancer, and emerging evidence indicates aberrant upregulation in CRC tissues and cell lines (Zhang W. et al., 2022; Ye et al., 2021; Cai et al., 2024). Collectively, these observations suggest a potential tumor-promoting role for CKMT2 in CRC, although its precise molecular mechanisms remain to be clarified.

PDE2A regulates cyclic nucleotide signaling through modulation of cAMP and cGMP levels (Limoncella et al., 2022). Its role in cancer appears context-dependent, with reported involvement in tumor growth and epithelial-mesenchymal transition across multiple malignancies, including colorectal cancer, melanoma, cholangiocarcinoma, pancreatic adenocarcinoma, and hepatocellular carcinoma (Zhao et al., 2021; Hiramoto et al., 2014; Zhang J. et al., 2022). While PDE2A expression is reduced in some tumor types, its activity has also been linked to prognostic stratification and therapeutic responsiveness in hepatobiliary and gastrointestinal cancers (Zhang J. et al., 2022; Li et al., 2021; Frolov et al., 2003). Moreover, PDE2A is a recognized pharmacological target of exisulind, highlighting its potential clinical relevance (Pusztai et al., 2003).

External validation in independent cohorts confirmed the opposing prognostic associations of these genes, with higher CKMT2 expression correlating with improved survival and elevated PDE2A predicting adverse outcomes. Immunotherapy analyses suggested that CKMT2 expression was higher among responders; however, these findings were derived from pan-cancer datasets and should be considered exploratory pending validation in CRC-specific immunotherapy cohorts.

This study relies primarily on publicly available transcriptomic datasets (TCGA, GTEx, GEO), which may introduce variability related to sample processing and clinical annotation. Although batch correction was applied to integrate multi-cohort data, residual effects cannot be fully excluded. Nevertheless, the reproducibility of findings across independent cohorts, their validation using single-cell RNA sequencing, and experimental confirmation in CRC cell lines and clinical specimens strengthen the robustness of the conclusions. Further functional and prospective clinical studies will be required to clarify the mechanistic roles of CKMT2 and PDE2A and their potential therapeutic implications.

## Conclusion

This study demonstrates a significant interaction between the TME and metabolism in CRC, emphasizing their roles in tumor progression and heterogeneity. CRC was classified into two distinct subtypes: C1 subtype, characterized by poorer prognosis, immune-mesenchymal features, and reliance on lipid metabolism; and C2 subtype, associated better prognosis, genomic instability, and enrichment in amino acid metabolism. Mutational profiling further distinguished these subtypes, with C1 enriched for TTN and BRAF mutations and C2 characterized by higher frequencies of APC and TP53 mutations. Additionally, PDE2A and CKMT2 emerged as key metabolic biomarkers, with PDE2A associated with poor prognosis through endothelial and stromal compartments and CKMT2 linked to favorable outcomes via malignant epithelial cells. Collectively, these findings enhance our understanding of CRC heterogeneity and highlight TMET-based stratification as a framework for prognostic assessment and the development of targeted therapeutic strategies.

## Data Availability

The original contributions presented in the study are included in the article/Supplementary Material, further inquiries can be directed to the corresponding authors.
